# Improved Breast Cancer Classification through Combining Transfer Learning and Attention Mechanism

**DOI:** 10.3390/life13091945

**Published:** 2023-09-21

**Authors:** Asadulla Ashurov, Samia Allaoua Chelloug, Alexey Tselykh, Mohammed Saleh Ali Muthanna, Ammar Muthanna, Mehdhar S. A. M. Al-Gaashani

**Affiliations:** 1 School of Communication and Information Engineering, Chongqing University of Posts and Telecommunications, Chongqing 400065, China; asadullahashur@gmail.com; 2 Department of Information Technology, College of Computer and Information Sciences, Princess Nourah bint Abdulrahman University, P.O. Box 84428, Riyadh 11671, Saudi Arabia; 3 Institute of Computer Technologies and Information Security, Southern Federal University, Taganrog 347922, Russia; tselykh@sfedu.ru (A.T.); muthanna@sfedu.ru (M.S.A.M.); 4 RUDN University, 6 Miklukho-Maklaya Street, Moscow 117198, Russia; muthanna.asa@spbgut.ru; 5 College of Computer Science and Technology, Chongqing University of Posts and Telecommunications, Chongqing 400065, China; mr.mehdhar@gmail.com

**Keywords:** breast cancer, CNN, attention mechanism, transfer learning, classification, malignant, benign, magnification level, histopathology image

## Abstract

Breast cancer, a leading cause of female mortality worldwide, poses a significant health challenge. Recent advancements in deep learning techniques have revolutionized breast cancer pathology by enabling accurate image classification. Various imaging methods, such as mammography, CT, MRI, ultrasound, and biopsies, aid in breast cancer detection. Computer-assisted pathological image classification is of paramount importance for breast cancer diagnosis. This study introduces a novel approach to breast cancer histopathological image classification. It leverages modified pre-trained CNN models and attention mechanisms to enhance model interpretability and robustness, emphasizing localized features and enabling accurate discrimination of complex cases. Our method involves transfer learning with deep CNN models—Xception, VGG16, ResNet50, MobileNet, and DenseNet121—augmented with the convolutional block attention module (CBAM). The pre-trained models are finetuned, and the two CBAM models are incorporated at the end of the pre-trained models. The models are compared to state-of-the-art breast cancer diagnosis approaches and tested for accuracy, precision, recall, and F1 score. The confusion matrices are used to evaluate and visualize the results of the compared models. They help in assessing the models’ performance. The test accuracy rates for the attention mechanism (AM) using the Xception model on the “BreakHis” breast cancer dataset are encouraging at 99.2% and 99.5%. The test accuracy for DenseNet121 with AMs is 99.6%. The proposed approaches also performed better than previous approaches examined in the related studies.

## 1. Introduction

Cancer, the primary cause of mortality in several nations, has become a significant global health concern. Cellulitis, or abnormal cell proliferation, is essential to developing malignancy. Tumors and cancers are characterized by the uncontrolled proliferation of cells, which results in the formation of aggregates or tumors in different organs. Lung, liver, colorectal, stomach, and breast cancers are the most prevalent forms of the disease [1]. Histopathology, the microscopic examination of biopsies by pathologists, is essential for diagnosing and comprehending the progression of organ cancer. Before microscopic examination, histological slides of tissue are prepared to facilitate examination. These transparencies display a variety of cells and tissue structures. Worldwide, breast cancer is the primary cause of mortality among female cancer patients. Its prevalence and fatality make it a significant global concern that affects a large number of women. Breast cancer is the most prevalent malignancy, accounting for approximately 14% of all cancers, with high mortality and morbidity rates among women worldwide. It increases the mortality rate among women, affecting approximately 2.1 million each year. It was estimated that 6,855,000 women would perish from breast cancer in 2020 [2]. Globally, death rates are on the rise, and they are significantly higher in wealthy nations. Tumors are masses that result from cancer’s aberrant cell division. A growth can be malignant (cancerous) or benign. Cancer of the breast is a malignant neoplasm that develops from breast cells.

According to clinical evidence, the survival rate of breast cancer patients can be significantly improved by early and accurate diagnosis. Although histopathological examination has long been regarded as the gold standard for breast cancer diagnosis, its accuracy can be affected by subjective factors and image texture quality [3]. This can result in incorrect diagnoses and unnecessary patient injury. In order to resolve these issues, it is anticipated that computer-assisted categorization of histological images will reduce pathologists’ burden and improve the accuracy and efficiency of pathological investigations. By leveraging technology, these advancements have the potential to yield substantial therapeutic and societal advantages in the field of breast cancer diagnosis. Deep learning techniques, specifically convolutional neural networks (CNNs), have demonstrated promise in medical image analysis tasks in recent years [4,5]. This article presents a novel method for analyzing breast cancer pathological images to improve feature extraction and diagnostic precision. The proposed method employs modified pre-trained CNN models and attention mechanisms to capture salient local features and derive an exhaustive representation of breast cancer images. Incorporating a channel attention module, which facilitates non-dimensionality reduction and local cross-channel interaction, is one of the main contributions of the proposed method. This module increases the CNN’s ability to extract pertinent information from pathological images by selectively concentrating on significant local features. In addition, the attention mechanism incorporates a spatial attention block that selectively highlights essential regions within the histopathological images. This attention mechanism assures the extracted breast cancer classification features are informative and highly pertinent. By focusing the network’s attention on critical regions, the proposed method accomplishes a robust global representation of features, enhancing overall diagnostic performance. Experiments are conducted on the BreakHis [6] breast cancer pathology dataset to evaluate the efficacy of the proposed method. The approach obtains comparable results to the most advanced models in the field, demonstrating its competitive performance. This paper proposes a novel breast cancer pathological image analysis approach incorporating attention mechanisms and modified pre-trained CNN models. The proposed method improves feature extraction by capturing salient local features and accentuating essential regions selectively, resulting in a comprehensive global representation of features. The experimental evaluation demonstrates the efficiency and competitiveness of the proposed breast cancer diagnosis method.

## 2. Related Works

A significant amount of studies have been conducted on classifying breast cancer images using deep learning methods. Two major approaches can be used to classify these investigations broadly [7]. The initial strategy utilizes representative CNN models as feature extractors and classifiers [8], followed by traditional machine learning models for classification [9].

Fatima et al. [10] review several works of literature that summarize and showcase prior studies focusing on machine learning algorithms employed in breast cancer prediction. Their work is a valuable resource for researchers seeking to analyze these algorithms and develop a fundamental understanding of deep learning research in this domain. Asri et al. [11] compare the efficacy of four machine learning algorithms using the Wisconsin Breast Cancer dataset: support vector machine (SVM), decision tree (DT), naïve Bayes (NB), and k-nearest neighbor (k-NN). The primary objective is to evaluate each algorithm’s accuracy, precision, sensitivity, and specificity in efficiently classifying the data. The experimental results demonstrate that SVM obtains the utmost accuracy of 97.13% with the lowest error rate. All investigations were conducted in a simulated environment using the WEKA data mining instrument. Liu et al. [12] proposed the AlexNet-BC model, a novel framework for breast pathology classification. They introduce an improved cross-entropy loss function that effectively handles overconfident low-entropy output distributions, ensuring more suitable predictions for uniform distributions. They conducted a comprehensive series of experiments using the BreaKHis, IDC, and UCSB datasets to validate their approach. The results consistently demonstrate that the proposed method achieved slightly better results across varying magnification levels. Ramesh et al. [13] incorporated the GoogLeNet architecture for segmentation purposes. The resulting segmentations serve as inputs to enhance the performance of classifiers such as SVM, DT, RF, and NB. This approach yields notable improvements in accuracy, Jaccard and dice coefficients, sensitivity, and specificity compared to traditional architectures. The authors’ proposed model achieves an accuracy score of 99.12%. Notably, it outperforms the AlexNet classifier by 3.78% in accuracy, and, on average, the improvement over existing techniques is 4.61%. Sharma and Kumar [14] explored the feasibility of leveraging a pre-trained Xception model for magnification-dependent breast cancer histopathological image classification, positioning it against conventional handcrafted methodologies. Notably, the Xception model using the ’radial basis function’ kernel, in tandem with an SVM classifier, showcased a superior and more consistent performance. Across magnification levels (40×, 100×, 200×, and 400×), accuracy figures of 96.25%, 96.25%, 95.74%, and 94.11% were achieved. Liew et al. [15] researched DLXGB (deep learning and eXtreme gradient boosting) and applied it to histopathology breast cancer images from the BreaKHis dataset to classify breast cancer. The method comprises data augmentation, stain normalization, and the use of a pre-trained DenseNet201 model for feature learning. Then, a gradient-boosting classifier is combined with these features. The classification task entails differentiating benign from malignant cases and classifying the images into eight non-overlapping/overlapping categories. The results demonstrate that DLXGB obtained a remarkable 97% accuracy for both binary and multi-class classification, superseding previous studies utilizing the same dataset. Atban at el. [16] concentrated on classifying breast cancer using optimized deep features. They used the ResNet18 architecture for feature extraction results in the production of deep features. Meta-heuristic algorithms, such as particle swarm optimization (PSO), atom search optimization (ASO), and the equilibrium optimizer (EO), are employed to improve the representativeness of these features further. Traditional ML algorithms are then used to evaluate the optimized deep features’ classification effect. The experimental analysis is conducted on the widely used benchmark dataset BreakHis. The results demonstrate that the proposed method, in particular, the features obtained from ResNet18-EO, obtains an impressive F-score of 97.75% when coupled with an SVM employing Gaussian and radial-based functions (RBF). Ayana et al. [17] proposed in breast cancer classification the multistage transfer learning (MSTL) algorithm, which employs three pre-trained models—EfficientNetB2, InceptionV3, and ResNet50—combined with three optimizers: Adam, Adagrad, and stochastic gradient descent (SGD). The dataset encompasses 20,400 cancer cell images, supplemented by 200 ultrasound images from Mendeley and 400 from the MT-Small-Dataset. Highlighting the versatility of MSTL, the ResNet50-Adagrad configuration yielded significantly higher test accuracy scores of 99 ± 0.612% on the Mendeley dataset and 98.7 ± 1.1% on the MT-Small-Dataset, demonstrating consistency across five-fold cross-validation. Wang et al. [18] proposed a new method for the automated diagnosis and staging of cancer based on image analysis and machine learning algorithms. They utilized the BreaKHis dataset and applied preprocessing procedures, such as color-to-grayscale conversion, thresholding, and filtering. Nuclei are segmented utilizing the distance transform and watershed algorithms. Two distinct techniques for feature extraction are investigated. An ensemble-tagged tree classifier obtains the most remarkable accuracy (89.7%) in binary classification (benign vs. malignant). An ensemble subspace discriminant classifier obtains an accuracy of 88.1% for multiclass classification. Dubey et al. [19] proposed a hybrid convolutional neural network architecture for classifying benign and malignant breast lesions in histopathological micrographs. The architecture incorporates a ResNet50 model that has been pre-trained with additional layers that include global average pooling, dropout, and batch normalization. The proposed method obtains state-of-the-art performance on the BreakHis data set, exceeding previous benchmarks. The model obtains an AUC of 0.94 and a precision of 98.7%, demonstrating its efficacy in managing the binary classification problem and overcoming target imbalance. The innovation of the proposed method resides in the strategic incorporation of ResNet50’s knowledge and training with the global average pooling layer. Singh et al. [20] proposed a hybrid deep neural network based on histopathological images for computer-assisted breast cancer diagnosis. The network integrates inception and residual blocks to capitalize on their respective benefits and achieve superior performance compared to existing algorithms. Two publicly available datasets, Breast Histopathology Images (BHI) and BreakHis, are used to train and validate the proposed method. With training precisions of 0.9642 for BreakHis and 0.8017 for BHI, the experimental results demonstrate the superiority of the proposed method. The model outperforms conventional deep neural networks and cutting-edge breast cancer detection techniques with accuracies of 0.8521 for BHI and 0.8080, 0.8276, 0.8655, and 0.8580 for distinct magnification levels (40×, 100×, 200×, and 400×) in the BreakHis dataset. Venugopal et al. [21] proposed a hybrid deep-learning model that combines Inspection-ResNetv2 and EfficientNetV2-S with ImageNet-trained weights to classify breast cancer histopathology images. Using the BreakHis and BACH datasets, the model is evaluated. The top layer is removed from both networks, and global average pooling, dense layers, dropout, and a final classification layer are added. The individual results from the Inspection-ResNetv2 and EfficientNetV2 models are contrasted with the model’s output. The final classification layer comprises four neurons for the BACH dataset and eight neurons for the BreakHis dataset. The experimental results demonstrate the proposed model’s efficacy, achieving an overall precision of 98.15 percent for the BACH dataset and 99.03 percent for the BreakHis dataset. Kumari et al. [22] introduced a transfer learning-based AI system for classifying breast cancer using histopathological images. The VGG-16, Xception, and Densenet-201 deep convolutional neural network architectures are used as base models in the transfer learning approach. Using the pre-trained base models, features are extracted from each test image, and the images are categorized as benign or malignant. The proposed system achieves high classification accuracies of 99.42% (IDC dataset) and 99.12% (BreaKHis dataset). Compared to extant breast cancer classification methodologies, the outcomes demonstrate superior performance. Additionally, the proposed system is independent of image magnification levels, which increases its utility and adaptability. Joshi et al. [23] proposed a deep CNN-based breast cancer detection framework. The BreakHis and IDC datasets are used to extract breast cancer-related information from histopathology images. Three pre-trained CNN models, EfficientNetB0, ResNet50, and Xception, are assessed, with the customized Xception model producing the best results. The BreakHis dataset’s 40 magnification images obtain an accuracy of 93.33 percent. The models are trained on 70 percent of the BreakHis dataset and validated on 30 percent of the remaining data. For regularization, data augmentation, dropout, and batch normalization are utilized. The enhanced Xception model is fine-tuned and tested on a subset of the IDC dataset, attaining an invasive ductal carcinoma detection accuracy of 88.08%. Adapting the pre-trained model to diverse classification tasks on the BreakHis and IDC datasets, this study demonstrates the efficacy of transfer learning. R Karthik et al. [24] introduced a novel classification method for breast cancer based on an ensemble of two deep convolutional neural network architectures, CSAResnet and DAMCNN, enhanced with Channel and Spatial attention. The framework extracts features from histopathology images in parallel using both architectures and employs ensemble learning to enhance performance. On the BreakHis dataset, the proposed method obtains a high classification accuracy of 99.55%. Zou et al. [25] proposed AHoNet with a channel attention module to capture local salient features within breast cancer pathological images, enhancing their discriminative power. Their method estimates second-order covariance statistics through matrix power normalization, providing a robust global feature representation. To assess the effectiveness of AHoNet, it was evaluated on two public breast cancer pathology datasets: BreakHis and BACH. The results demonstrate AHoNet’s competitive performance, achieving optimal patient-level classification accuracies of 99.29% on BreakHis and 85% on BACH. These findings underscore the potential of attention mechanisms in enhancing breast cancer classification, aligning with the objectives of the proposed research, which combines transfer learning and attention mechanisms for breast cancer classification. Jadah et al. [26] introduced a breast cancer classification model based on the AlexNet convolutional neural network. The model is applied to histopathological images from the BreakHis dataset to diagnose breast cancer. Various modifications to the parameters and data are implemented to enhance the model’s ability to recognize and classify input images as benign or malignant tumors. By optimizing the training frequency and balancing the training data, a classification accuracy of up to 96% is achieved. This research paper reviewed various approaches and advancements in breast cancer detection and classification, which are highlighted by the related works. Various techniques, such as machine learning algorithms, deep learning models, and ensemble methods, have been utilized to accurately analyze histopathological images and diagnose breast cancer. Transfer learning, data augmentation, and feature extraction techniques have been extensively used to improve classification model performance. The studies highlight the significance of image normalization, segmentation, and feature selection for increasing the accuracy of breast cancer classification systems. In addition, the use of publicly accessible datasets, such as BreakHis and IDC, has allowed researchers to compare and evaluate the efficacy of various methods. The related works demonstrate the potential of computational approaches to aid medical professionals in the early detection and accurate diagnosis of breast cancer, paving the way for enhanced patient outcomes and individualized treatment strategies. Several research voids exist in the classification of breast cancer that must be addressed. In machine learning-based approaches, there is a reliance on manual feature extraction, which can be time-consuming and subjective. To combat this, it is necessary to investigate methods of deep learning that can autonomously learn pertinent features from histopathology images. An additional area for improvement is the imbalance in training datasets, which can result in biased predictions. Effective data augmentation strategies are required to represent all classes adequately. In addition, existing methods frequently employ single-path deep learning architectures, which may lead to a more significant number of false positives and false negatives. Consider utilizing sophisticated techniques such as feature fusion and ensembling to increase classification accuracy. Motivated by these gaps, this paper proposes a novel classification method for breast cancer that tackles these limitations and enhances diagnostic accuracy.

## 3. Materials and Methods

This section presents the proposed image classification architecture for breast cancer using histopathology images. In addition, we investigate the specifics and fundamentals of the essential methods used in this study to assess their effect on medical image categorization.

**Figure 1:**
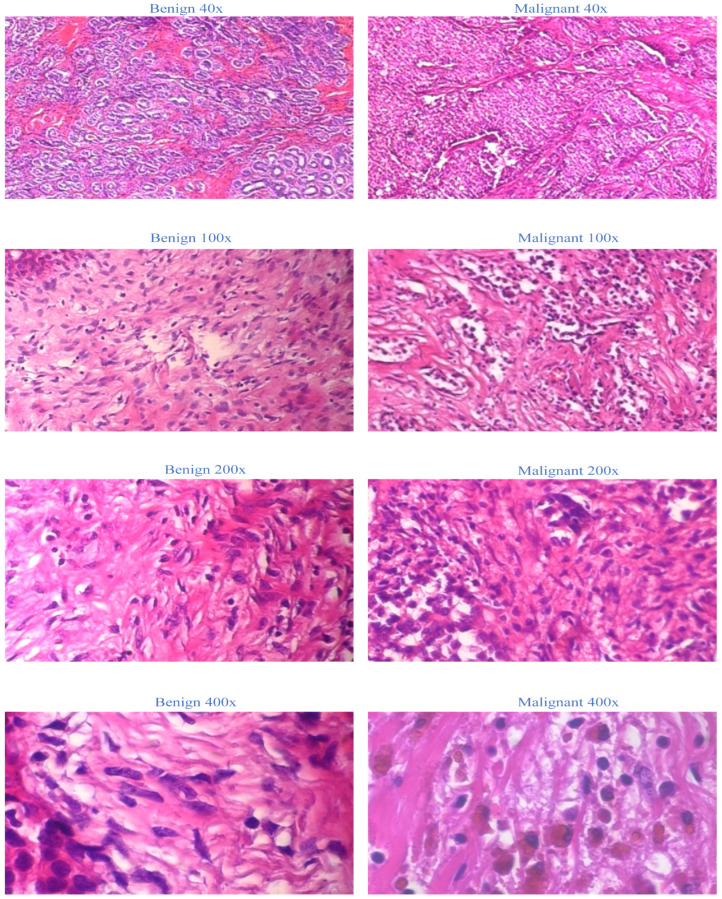
Representation of BreakHis dataset at four magnifications.

The BreakHis dataset is selected as the sole dataset for evaluating our proposed models. This extensive collection of non-full-field breast cancer histopathology images is chosen due to its widespread recognition and accessibility. We establish a firm foundation for our medical application by utilizing the BreakHis dataset. This decision ensures the validity and generalizability of our proposed model, as the BreakHis dataset is representative of a typical dataset for image multiclassification tasks in breast cancer pathology. The 2016-created BreakHis dataset is available online via the breast cancer database. It consists of 7909 histopathological images of breast tumors from 82 patients. The dataset comprises 2480 benign tumor images (fibroadenoma, adenoma, tubular adenoma, and trichome tumors) and 5429 malignant tumor images (lobular carcinoma, ductal carcinoma, papillary carcinoma, and mucinous carcinoma) obtained at four magnifications: 40×, 100×, 200×, and 400×. In the breast tumor pathology section, each image from the BreakHis database is displayed with a 700-460-pixel resolution and RGB color format. Figure 1 depicts breast tissue segments, including malignant tumors, from the BreakHis dataset at four magnifications.

The dataset is divided into training and test sets based on the magnifications for the purpose of breast cancer prediction. Table 1 depicts the distribution of samples across various magnification levels (40×, 100×, 200×, and 400×) and the corresponding division into training and testing subsets. The dataset is separated into training and test sets for each magnification level. The training set is used to train models for predicting breast cancer, while the testing set is used to evaluate the efficacy of the trained models. This division of the dataset, systematically based on the magnification level, guarantees that the trained models are evaluated using independent and representative samples from each magnification level. This enables a comprehensive evaluation of the models’ performances at various magnification levels, contributing to the robustness and generalizability of the research findings.

**Figure 2:**
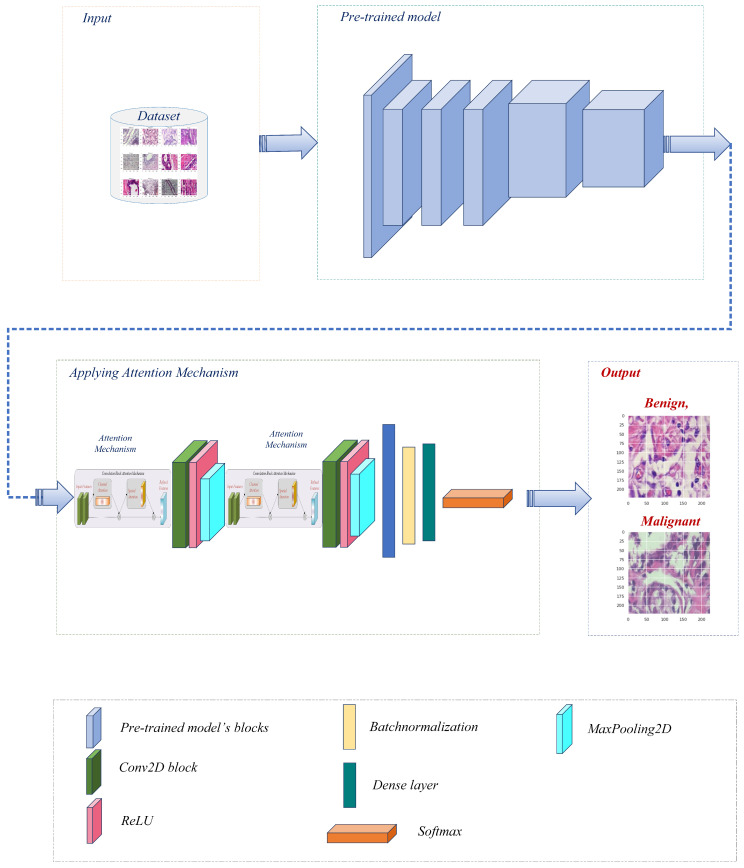
The proposed breast cancer detection workflow utilized a pre-trained model with an attention mechanism.

Before further modifications are made to several pre-trained models [27], namely Xception, VGG16, ResNet50, MobileNet, and DenseNet121, the BreakHis dataset is preprocessed. After each individual model, the convolutional layer is connected to construct a modified architecture for each model. Figure 2 represents the overall pipeline of the proposed system for breast cancer classification in this study.

The preprocessing stages for the BreakHis dataset likely included resizing the images to a consistent size, normalizing pixel values, and possibly applying additional transformations to improve the dataset’s quality and consistency.

After preprocessing, each pre-trained model is modified by adding a convolutional layer. This additional layer permits further extraction and refinement of BreakHis-specific features. By connecting the conv layer after each model individually, the modified architectures can effectively capture and learn from the dataset’s pertinent patterns and characteristics. The architecture uses pre-trained models as its backbone, leveraging prior knowledge to extract meaningful and representative features from the input images. This incorporation enables the model to leverage the ImageNet dataset’s extensive feature representations. Following that, convolutional layers, max-pooling layers, channel attention layers, and spatial attention layers are included in the architecture. These components collaborate to improve the extraction of salient image features and highlight pertinent image regions. The extracted multidimensional features are then transformed into a compact 1D vector by a flattened layer, which is followed by batch normalization and a dense layer with softmax activation for efficient classification. The model is compiled utilizing the binary cross-entropy loss function and the well-known Adam optimizer [28] with a variable learning rate. We prioritize optimal performance and resource allocation by incorporating memory management techniques. It helps mitigate potential memory leaks, reduce memory usage, and enhance the overall efficiency of the deep learning model.

### 3.1. Attention Mechanism

The Convolutional Block Attention Module (CBAM) [29]: In our research, we applied the CBAM to address the challenges posed by limited data and computational constraints in breast cancer histopathology image classification. The CBAM module selectively highlights relevant features and suppresses extraneous ones, thereby improving the performance of the model [30].

The Channel Attention Module: The channel attention module focuses on capturing channel interactions. Given the input feature map *F*, we perform global average pooling and global max-pooling to obtain average-pooled ($FC_{\mathrm{Avg}}$) and max-pooled ($FC_{\mathrm{Max}}$) features, respectively. These features are then individually passed through a multilayer perceptron (MLP), and the resulting elements are added element-wise. This process generates the channel attention map (Mc(F)), representing inter-channel relationships within the input feature map.

To incorporate the channel attention map, we perform element-wise multiplication between the input feature map (*F*) and the channel attention map (Mc(F)), which can be expressed as:

(1) $$FC=\sigma (M\left(FC_{\mathrm{Max}}\right)+M\left(FC_{\mathrm{Avg}}\right))⊗F,$$

where σ denotes the sigmoid function.

The Spatial Attention Module: The spatial attention module aims to leverage inter-spatial connections between features. We apply average pooling and max-pooling along the channel axis to generate two feature maps, $FS_{\mathrm{Avg}}$ and $FS_{\mathrm{Max}}$. These feature maps are then concatenated along the channel axis and processed through a convolution layer to generate a spatial attention map (Ms(F)) with dimensions H×W×1. To integrate the inter-spatial connections, the spatial attention map (Ms(F)) is element-wise multiplied with the input feature map (FC), resulting in the revised feature map FCS with dimensions H×W×C. This process can be expressed as:

(2) $$FCS=\sigma (\mathrm{Conv}\left(\mathrm{Cat}\left[FS_{\mathrm{Max}};FS_{\mathrm{Avg}}\right]\right))⊗FC,$$

where Conv() represents a convolution process with a 7×7 filter size.

**Figure 3:**
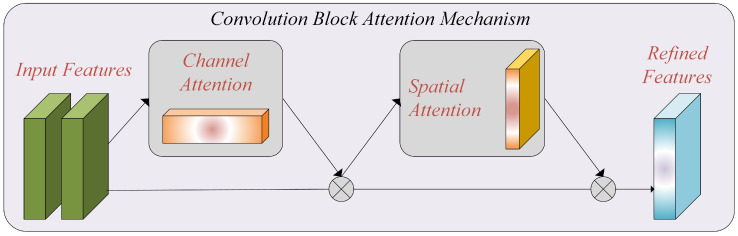
The attention mechanism, which includes channel and spatial attention blocks.

By incorporating the CBAM module into our model, we enhance the ability to capture essential features and improve the performance of breast cancer histopathology image classification. Figure 3 represents the attention mechanism used in this study.

### 3.2. Feature Extraction

In our proposed method for histopathology image classification on the BreakHis dataset, we integrate attention mechanisms into the feature extraction stage. These mechanisms are intended to improve the extraction of salient image features and highlight relevant regions, thereby enhancing the model’s discriminative ability [31]. This section details the feature extraction process of incorporating attention mechanisms. The feature extraction phase begins by employing pre-trained models as the foundation of our architecture. We can extract meaningful and representative features from input images by leveraging prior knowledge from models trained on the ImageNet dataset. This technique takes advantage of the extensive feature representations acquired from a large and diverse dataset. Our proposed architecture includes convolutional layers, max-pooling layers, channel attention layers, and spatial attention layers that collaborate to refine the derived characteristics. The convolutional layers employ filters to capture various image patterns, whereas the max-pooling layers downsample the feature maps to preserve the most pertinent data. The attention mechanisms are crucial to our feature extraction procedure. The channel attention layers permit the model to dynamically adjust the significance of various channels within the feature maps. The model can extract more discriminative features and suppress irrelevant data by concentrating on the most informative channels. Similarly, the spatial attention layers enable the model to highlight pertinent image regions by adaptively weighting the spatial locations within the feature maps. This mechanism focuses the model’s attention on the most informative regions, which improves the localization and representation of pertinent image features. The incorporation of attention mechanisms offers several benefits. First, it enhances the model’s ability to capture fine-grained details and intricate image patterns. By selectively focusing on the most informative channels and spatial locations, the model becomes more sensitive to pertinent characteristics, contributing to accurate classification. The attention mechanisms improve the interpretability of the decision-making process of the model. We gain insight into which image regions and features influence the model’s predictions by highlighting the significant regions and channels. This interpretability can be advantageous in applications that require transparency and explicability. The attention mechanisms contribute to the overall efficacy of the feature extraction process. The model can reduce computational redundancy and derive more compact and discriminative representations by focusing on pertinent image regions and features. This efficacy results in quicker inference times and enhanced utilization of computational resources. The attention mechanisms improve the extraction of prominent image features, enhance interpretability, and optimize computational efficiency. By incorporating attention mechanisms, our model is able to effectively capture pertinent information and accomplish enhanced performance in image classification tasks using the BreakHis dataset.

### 3.3. Classification

Following the feature extraction phase, the extracted features are forwarded to the classification phase [32], where attention mechanisms are employed to refine the feature representations further and enhance the model’s discriminative power. During the classification phase, we employ attention mechanisms to dynamically weight the importance of distinct features within the extracted representations. This enables the model to concentrate on the most pertinent information for accurate prediction. We apply attention layers to the extracted feature representations to integrate attention mechanisms. These layers determine the attention weights for each feature based on their importance to the classification assignment. The attention weights are then applied to the features, accentuating those most informative while downplaying those less pertinent. Our model acquires several benefits by integrating attention mechanisms into the classification stage. First, it enables the model to concentrate on most informative discriminative characteristics for the classification assignment. This selective focus improves the model’s robustness and precision by minimizing the influence of irrelevant or chaotic features. Attention mechanisms improve the interpretability of the classification procedure. By displaying the attention weights, we can determine which features influence the model’s decision-making. This interpretability permits us to gain insight into the model’s logic and provides a method for validating its predictions. It can mitigate the effects of class disparity by concentrating on underrepresented classes. The model can enhance its performance on these classes by allocating greater attention weights to minority-class samples, resulting in a more accurate and balanced classification. Incorporating attention mechanisms into the classification phase improves the model’s discriminative ability, interpretability, and robustness [33]. By dynamically balancing the significance of features, the model can prioritize pertinent information and make more accurate predictions. Incorporating attention mechanisms into the classification stage enhances the overall performance of our proposed method for image classification on the BreakHis dataset.

## 4. Results

This section evaluates and analyzes the proposed CNN models with attention mechanisms for histopathology image classification on the BreakHis dataset. The model’s performance is evaluated based on classification accuracy, precision, recall, and F1-score. We conducted the same experimental configuration with other prominent CNN architectures, including Xception, VGG16, ResNet50, MobileNet, and DenseNet121. Regarding accuracy and other evaluation metrics, our proposed model with attention mechanisms consistently outperformed these architectures, demonstrating its efficacy in breast cancer classification.

Table 2 displays the evaluation of the performance of the proposed models with attention mechanisms on the BreakHis dataset at different magnification levels (40×, 100×, 200×, and 400×). Included in the evaluation metrics are validation accuracy and validation loss. The Xception model obtained high accuracy rates at all magnification levels, ranging from 98.5% to 99.5%, as shown in Table 1. The loss values are consistently modest, ranging between 0.02 and 0.04. This indicates that the model can accurately classify breast histopathology images with attention mechanisms. Compared to Xception, the VGG16 model displayed accuracy rates from 92.8% to 97.2%. The model obtained acceptable performance despite the dataset’s complexity. ResNet50 exhibited consistent performance across magnification levels, with 98.0% and 98.8% accuracy rates and minimal loss values between 0.05 and 0.09. This demonstrates the robustness and efficacy of the model in breast cancer classification. The MobileNet model obtained accuracy rates ranging from 92.4% to 99.2%, which are deemed satisfactory. However, its loss values are significantly greater than those of other models, ranging from 0.05 to 0.26. It may require additional optimization and finetuning to achieve optimal performance. DenseNet121 demonstrated a competitive level of performance, with accuracy rates ranging from 95.5% to 99.5%. The model consistently obtained low loss values varying from 0.02 to 0.12, demonstrating its classification accuracy. The experimental results demonstrate that incorporating attention mechanisms into proposed breast cancer classification models is effective. Xception and DenseNet121 demonstrated especially promising results. These findings validate the benefits of attention mechanisms in enhancing the ability of models to focus on pertinent features and regions, which contributes to enhanced classification performance.

**Figure 4:**
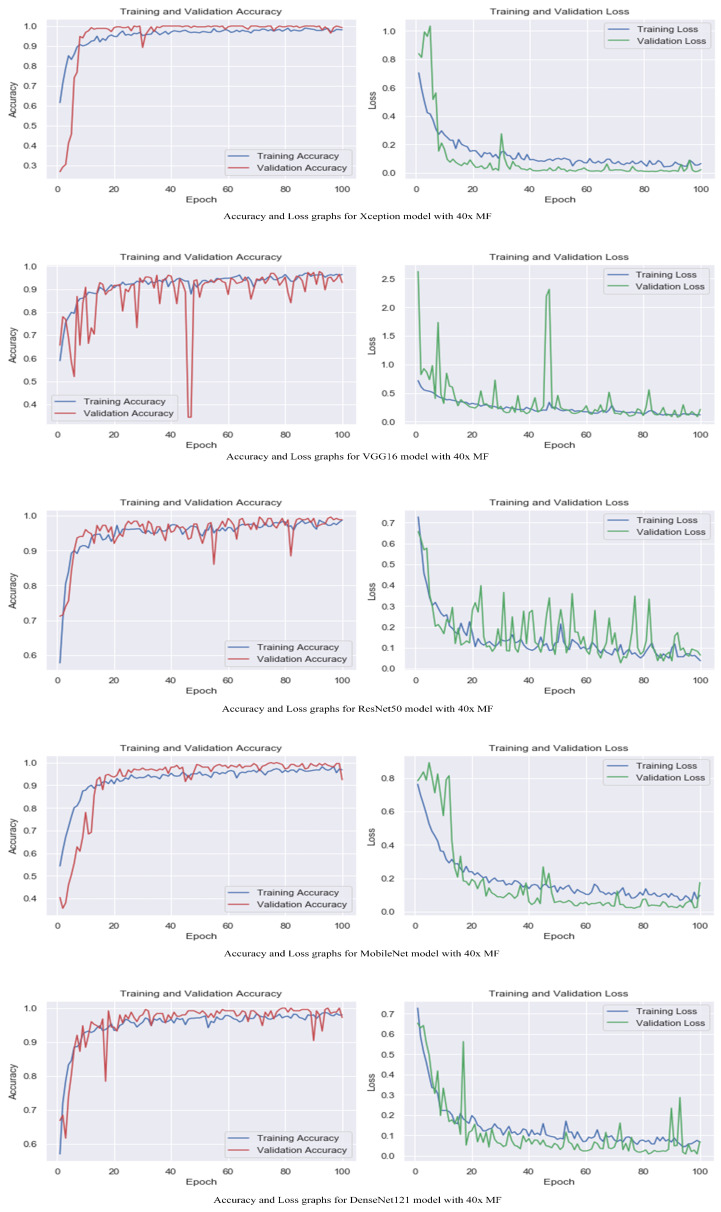
Accuracy and loss graphs for proposed models with 40× magnification level.

**Figure 5:**
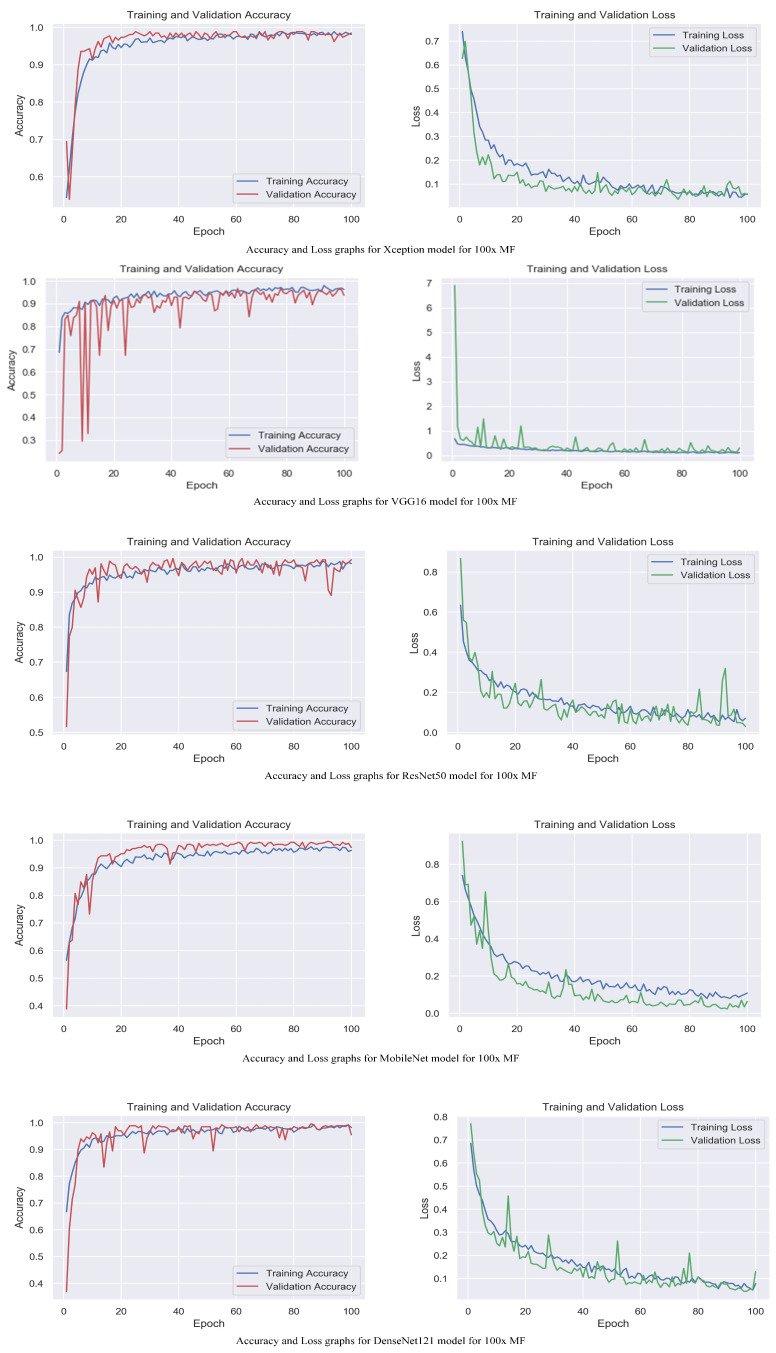
Accuracy and loss graphs for proposed models with 100× magnification level.

**Figure 6:**
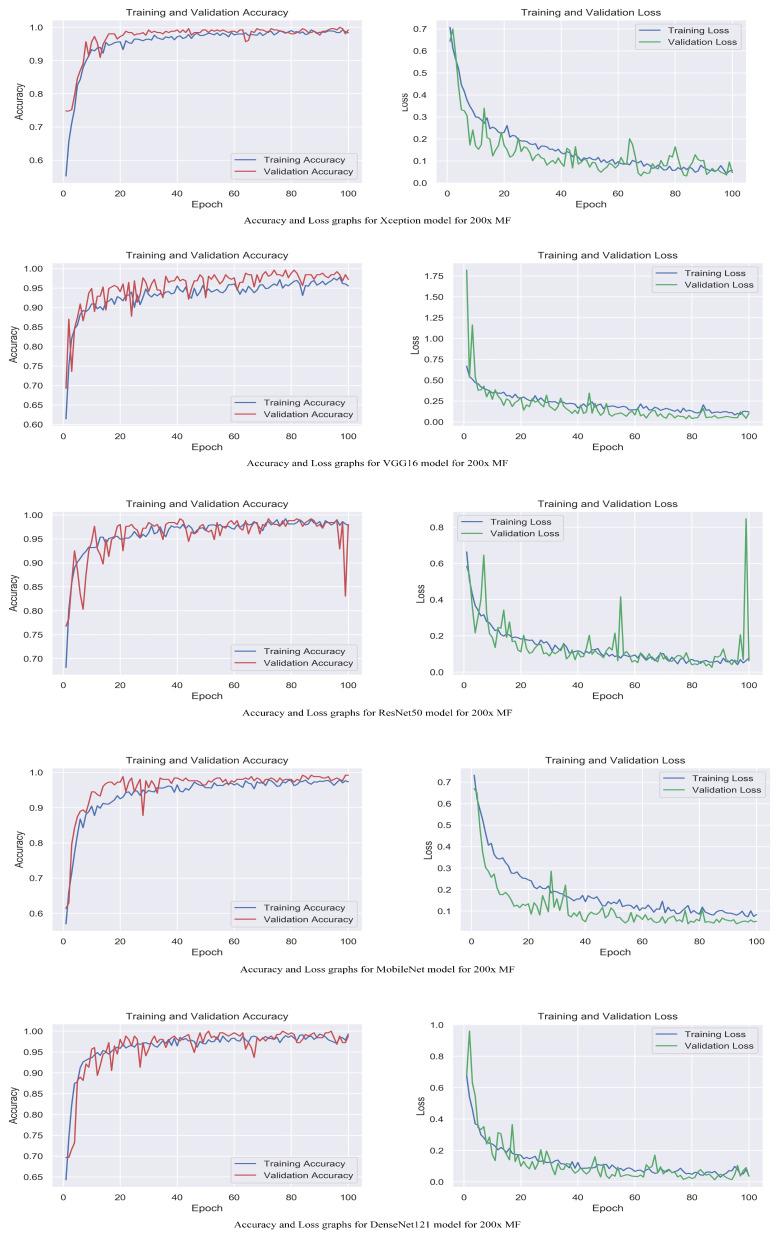
Accuracy and loss graphs for proposed models with 200× magnification level.

**Figure 7:**
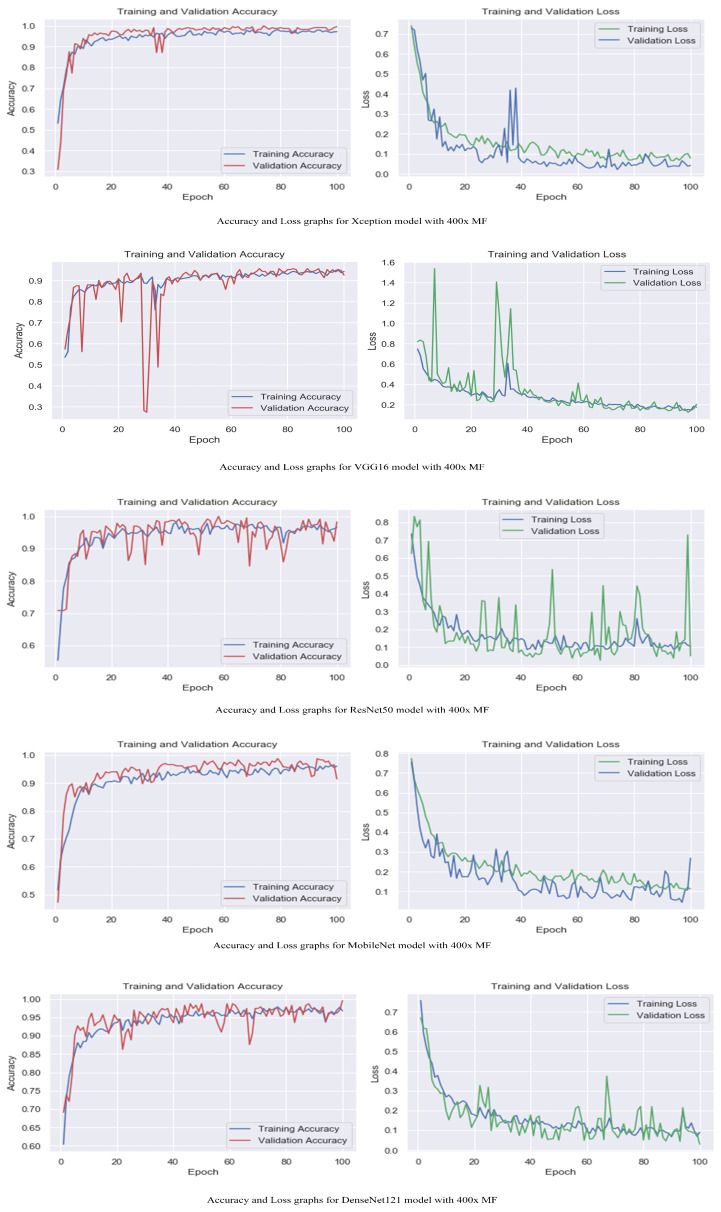
Accuracy and loss graphs for proposed models with 400× magnification level.

The accuracy and loss graphs among magnification levels are to visually illustrate in Figure 4, Figure 5, Figure 6 and Figure 7 the performances of the proposed models at different magnification levels. These graphs provide an intuitive representation of how the models perform in terms of accuracy and loss as the magnification level of the images varies. They provide a visual understanding of how the models’ accuracy and loss values change as the level of magnification increases or decreases. Accuracy and loss graphs across magnification levels offer an informative visual representation of the model’s performance, enhancing the clarity and comprehensibility of the research findings.

The precision performance results presented in this paragraph are based on our experiments with the proposed models and the BreakHis dataset. The macro average and weighted average metrics provide valuable insight into the precision performance of the models across all magnification levels, considering both equal and variable class distributions. In terms of precision, the efficacy of the proposed models at the 40×, 100×, 200×, and 400× magnification levels are evaluated. Precision measures a model’s ability to classify positive instances correctly. Due to providing a thorough evaluation, we utilize macro average metrics to assess various performance metrics. The macro average calculates the average value of these metrics across all magnification levels, assigning equal weight to each level. This approach provides an overall performance measure that treats all magnification levels equally, regardless of class distribution. It is useful for evaluating the models’ general performance without considering class imbalances at different magnification levels. In contrast, the weighted average metrics consider the class distribution for each magnification level. They calculate the average value of the metrics while giving more weight to magnification levels with larger class sizes. This approach offers a more comprehensive evaluation of the models’ performance, considering the varying class distributions across magnification levels. By giving appropriate weight to each magnification level based on its class distribution, the weighted average metrics provide a more representative assessment that avoids bias toward the most frequent class and reflects the true performance across different magnification levels.

Table 3 shows that Xception consistently obtained the highest precision performance across all magnification levels, with scores ranging from 86.1% to 90.4% based on the macro average. The VGG16 model’s precision performance is marginally inferior, with scores ranging from 81.3% to 87.2%. Xception and ResNet50 maintained their superiority, obtaining precision scores from 86.3% to 88.2% and from 89.0% to 88.2%, respectively, when considering the weighted average. The precision ratings for VGG16 and MobileNet ranged from 85.4% to 87.2% and 86.1% to 88.2%, respectively. These results demonstrate that the precision performance of the proposed models varies with the magnification level. Xception and ResNet50 have consistently demonstrated greater precision than VGG16 and DenseNet121. The evaluation of precision using both macro average and weighted average metrics provides a comprehensive comprehension of the precision capabilities of the models for breast cancer classification tasks at varying magnification levels.

The recall performance results are present based on experiments conducted on the BreakHis dataset using the proposed attention mechanism models. The macro average and weighted average metrics provide valuable insight into the overall recall performance of the models across all magnification levels. Table 4 displays the recall performance of the proposed models with the attention mechanism at various magnification levels. Recall, also known as sensitivity or true positive rate, quantifies the model’s ability to accurately identify positive instances (for example, correctly classifying malignant cells in the BreakHis dataset). The overall recall performance of the models across all magnification levels is evaluated using macro average and weighted average metrics. The macro average computes the average recall value across all magnification levels, considering each level equally. The weighted average considers the class distribution at each magnification level, providing a more accurate evaluation by giving greater weight to magnification levels with larger class sizes. According to Table 4, Xception achieved the highest recall performance across all magnification levels, with macro average scores between 84.1% and 88.2%. The macro average recall performance of VGG16 ranged between 74.6% and 83.4%. ResNet50 exhibited excellent recall performance, with macro average scores ranging from 85.5 to 87.7 percent. The MobileNet model exhibits variable recall performance, with macro average scores ranging between 80.1% and 88.5%. The recall performance of DenseNet121 is relatively consistent, with macro average scores ranging from 84.4% to 86.9%. Based on the weighted average, Xception and ResNet50 maintain their superiority, with recall scores spanning from 87.3% to 90.4% and from 86.3% to 90.1%, respectively. The weighted average recall scores for the VGG16 and MobileNet models range between 81.2% and 86.1% and between 86.3% and 87.6%, respectively. The weighted average recall scores for DenseNet121 range between 86.1% and 88.6%. These results demonstrate that the recall performance of the proposed models with an attention mechanism varies across various magnification levels. Xception and ResNet50 demonstrated consistently superior recall performance, whereas VGG16 and DenseNet121 demonstrated relatively inferior performance. The evaluation of recall using both macro average and weighted average metrics provides a comprehensive insight into the ability of models.

The F1-score performance results presented in this section are based on experiments performed on the BreakHis dataset using the proposed pre-trained models with an attention mechanism. The macro average and weighted average metrics provide valuable insight into the models’ overall F1-score performance across magnification levels, considering both equal and variable class distributions. Table 5 illustrates the F1-score performances of the proposed pre-trained models with attention mechanisms at varying magnification levels. The F1 score is a metric that integrates precision and recall to evaluate a model’s performance in binary classification tasks. Similar to the preceding sections, macro average and weighted average metrics are used to evaluate the overall F1-score performance of the models across all levels of magnification. The macro average computes the average F1-score across all magnification levels, considering each level equally. The weighted average considers the class distribution at each magnification level, providing a more accurate evaluation by giving greater weight to magnification levels with larger class sizes. According to the data presented in Table 5, Xception demonstrated strong F1-score performance at all magnification levels, with macro average scores ranging from 86.5% to 89.1%. ResNet50 also demonstrates impressive F1-score performance, with macro average scores spanning between 87.3% and 88.3%. MobileNet exhibited relatively consistent F1-score performance, with macro average scores ranging between 83.5% and 88.5%. The F1-score performance of DenseNet121 is stable, with macro average scores ranging from 85.3% to 87.6%. However, VGG16 demonstrated inferior F1-score performance to the other models, with average macro scores ranging between 77.9% and 84.6%.

Xception and ResNet50 maintain their superiority, with F1-score scores spanning from 87.6% to 90.3% and from 87.3% to 90.2%, respectively, when the weighted average is considered. MobileNet demonstrated weighted average F1-score scores ranging from 85 to 88 percent, whereas DenseNet121 demonstrated scores ranging from 86.1% to 88.5%. VGG16 demonstrated a weighted average F1 score, with scores ranging from 83.2% to 88.3%. Evaluating the F1-score using macro average and weighted average metrics comprehensively comprehends the models’ overall performance in binary classification tasks at varying magnification levels. Xception and ResNet50 consistently exhibited superior F1-score performances, whereas VGG16 demonstrated relatively inferior performance. The F1-score evaluation allows us to assess the models’ ability to reconcile precision and recall when identifying positive instances at different magnification levels.

**Figure 8:**
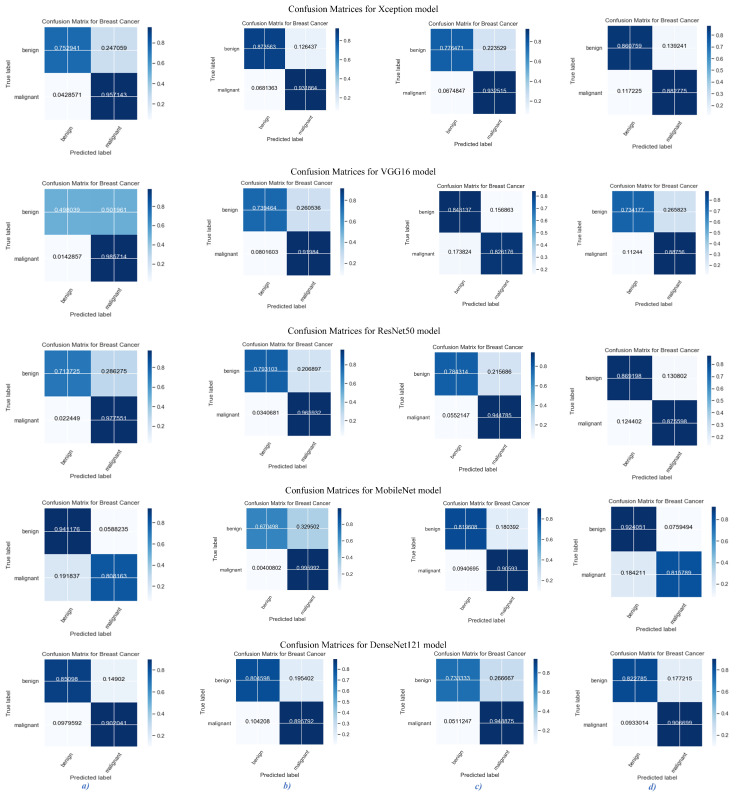
Confusion matrices for proposed models with four magnification levels. Column (**a**) 40×, Column (**b**) 100×, Column (**c**) 200×, Column (**d**) 400×.

We have presented the outcomes of our proposed models for breast cancer classification using histopathological images at four distinct levels of magnification: 40×, 100×, 200×, and 400×. To assess the performance of these models, we employed confusion matrices, which provide a comparison of the model’s predictions to the actual disease labels. Analyzing the confusion matrices for the proposed pre-trained models with attention mechanisms in breast cancer classification provides valuable insight into their performance. The true positive (TP), true negative (TN), false positive (FP), and false negative (FN) rates provide a comprehensive comprehension of the models’ ability to classify malignant and benign samples accurately. Figure 8 represents confusion matrices across all magnification levels for the proposed models. In this figure, Xception consistently demonstrates higher TP and TN rates than other models evaluated, indicating its superior performance in identifying malignant and benign samples reliably. ResNet50 demonstrates competitive TP and TN rates, indicating its efficacy in classification tasks. VGG16 and MobileNet, on the other hand, have relatively lesser TPR and TNR rates, indicating that their classification performance has room for improvement.

The disorientation matrices are organized into four columns, one for each level of magnification. Column “*(a)*” indicates a magnification level of 40×, column “*(b)*” indicates a magnification level of 100×, column “*(c)*” indicates a magnification level of 200×, and column “*(d)*” indicates a magnification level of 400×.

These outcomes are consistent with the previous results of our proposed system, in which Xception and ResNet50 outperformed VGG16 and MobileNet in terms of accuracy, precision, recall, and F1-score. Consequently, the analysis of the confusion matrices further validates the superiority of the Xception and ResNet50 models in classifying breast cancer samples accurately.

## 5. Conclusions

This study investigates the efficacy of pre-trained models with attention mechanisms for breast cancer classification at various magnification levels. Xception, VGG16, ResNet50, MobileNet, and DenseNet121 are evaluated using accuracy, loss rates, precision, recall, and F1-score metrics among macro and weight averages. The obtained results indicate that the choice of magnification level substantially affects classification performance. Different magnification levels resulted in models with varying degrees of accuracy, loss, precision, recall, and F1-score. This is due to differences in image quality, resolution, and the presence of distinct histopathological characteristics at each magnification level. Therefore, it is essential to consider the appropriate magnification level when designing and implementing classification systems for breast cancer. Compared to other models, Xception consistently demonstrated superior performance across most evaluation metrics, including accuracy, precision, recall, and F1-score. ResNet50 and DenseNet121 also performs competitively, whereas VGG16 and MobileNet, produce relatively inferior results. These results highlight the significance of selecting a suitable pre-trained model for breast cancer classification tasks, considering the model’s ability to extract pertinent features and recognize intricate patterns within the histopathological images. The incorporation of attention mechanisms into the models proved advantageous, as this improved the extraction of salient features and the overall classification performance. The attention mechanisms enabled the models to focus on significant regions and emphasize pertinent information, thereby contributing to more precise and robust predictions. This demonstrates the significance of attention mechanisms in deep learning-based medical image analysis and their potential to improve breast cancer detection and diagnosis. This study emphasizes the significance of incorporating magnification levels and attention mechanisms into breast cancer classification models. The findings contribute to the existing corpus of knowledge in the field of medical image analysis and offer valuable insights to breast cancer diagnosis researchers and practitioners. To further improve the performance of breast cancer classification models, additional research is encouraged to investigate other advanced techniques, evaluate larger data sets, and incorporate evaluation metrics.

## Figures and Tables

**Table 1:** Splitting the dataset among the magnification levels into training and testing.

Magnification Level	40×		100×		200×		400×		Total
**Splitting to Train and Test Set**	**Training**	**Testing**	**Training**	**Testing**	**Training**	**Testing**	**Training**	**Testing**	**All**
Benign	370	255	383	261	368	255	351	237	2480
Malignant	880	490	938	499	901	489	814	418	5429
Total	1250	745	1321	760	1269	744	1165	655	7909

**Table 2:** Accuracy rates and loss of the proposed models with attention mechanism.

Method\Magnification Level	40×		100×		200×		400×	
	Accuracy	Loss	Accuracy	Loss	Accuracy	Loss	Accuracy	Loss
Xception	99.2	0.02	98.5	0.05	99.2	0.04	99.5	0.04
VGG16	92.8	0.21	93.6	0.29	97.2	0.10	94.8	0.15
ResNet50	98.8	0.06	98.1	0.09	98.0	0.06	98.2	0.05
MobileNet	92.4	0.17	96.2	0.06	99.2	0.05	91.4	0.26
DenseNet121	97.2	0.07	95.5	0.12	98.8	0.11	99.6	0.02

**Table 3:** Precision performances of the proposed models with attention mechanism across magnification levels.

Magnification Level\Model	40×		100×		200×		400×	
	Macro Average (%)	Weighted Average (%)	Macro Average (%)	Weighted Average (%)	Macro Average (%)	Weighted Average (%)	Macro Average (%)	Weighted Average (%)
Xception	89.2	89.1	90.4	90.3	86.3	87.5	86.3	88.2
VGG16	87.1	84.0	85.4	86.3	81.3	84.2	85.6	87.2
ResNet50	91.7	88.2	91.6	90.1	89.0	89.4	86.3	88.1
MobileNet	87.7	89.5	90.2	88.2	86.6	88.3	86.1	88.4
DenseNet121	86.5	87.4	85.4	86.6	88.2	88.5	87.3	88.1

**Table 4:** Recall performances of the proposed models with attention mechanism across magnification levels.

Model\Magnification Level	40×		100×		200×		400×	
	Macro Average (%)	Weighted Average (%)	Macro Average (%)	Weighted Average (%)	Macro Average (%)	Weighted Average (%)	Macro Average (%)	Weighted Average (%)
Xception	84.1	88.3	88.2	90.4	85.6	87.5	87.3	87.6
VGG16	74.6	82.5	83.4	86.1	83.0	83.3	81.2	84.5
ResNet50	85.5	89.4	87.7	90.1	86.3	89.1	87.2	87.1
MobileNet	88.4	88.2	80.1	86.3	86.2	88.2	88.5	87.6
DenseNet121	86.8	87.3	85.2	86.1	84.4	88.3	86.9	88.6

**Table 5:** F1-score performances of the proposed models with attention mechanism across magnification levels.

Model\Magnification Level	40×		100×		200×		400×	
	Macro Average (%)	Weighted Average (%)	Macro Average (%)	Weighted Average (%)	Macro Average (%)	Weighted Average (%)	Macro Average (%)	Weighted Average (%)
Xception	86.5	88.3	89.1	90.3	85.7	87.6	87.2	88.1
VGG16	77.9	88.3	84.6	86.6	82.4	83.2	82.1	84.8
ResNet50	87.3	88.5	88.3	90.2	87.8	89.4	87.3	87.7
MobileNet	87.8	88.4	83.5	85.6	86.2	88.5	86.1	87.4
DenseNet121	86.7	87.3	85.5	86.1	85.3	87.4	87.6	88.5

## Data Availability

The data are contained within the article and/or available from the corresponding author upon reasonable request.
